# Single-Cell Regulatory Network Inference and Clustering Identifies Cell-Type Specific Expression Pattern of Transcription Factors in Mouse Sciatic Nerve

**DOI:** 10.3389/fncel.2021.676515

**Published:** 2021-12-08

**Authors:** Mingchao Li, Qing Min, Matthew C. Banton, Xinpeng Dun

**Affiliations:** ^1^Department of Neurology, The Affiliated Huai’an No. 1 People’s Hospital of Nanjing Medical University, Huai’an, China; ^2^School of Pharmacy, Hubei University of Science and Technology, Xianning, China; ^3^School of Biomedical Science, Faculty of Health, University of Plymouth, Plymouth, United Kingdom; ^4^The Co-innovation Center of Neuroregeneration, Nantong University, Nantong, China

**Keywords:** peripheral nerves, transcription factors, expression, scRNA-seq, SCENIC

## Abstract

Advances in single-cell RNA sequencing technologies and bioinformatics methods allow for both the identification of cell types in a complex tissue and the large-scale gene expression profiling of various cell types in a mixture. In this report, we analyzed a single-cell RNA sequencing (scRNA-seq) dataset for the intact adult mouse sciatic nerve and examined cell-type specific transcription factor expression and activity during peripheral nerve homeostasis. In total, we identified 238 transcription factors expressed in nine different cell types of intact mouse sciatic nerve. Vascular smooth muscle cells have the lowest number of transcription factors expressed with 17 transcription factors identified. Myelinating Schwann cells (mSCs) have the highest number of transcription factors expressed, with 61 transcription factors identified. We created a cell-type specific expression map for the identified 238 transcription factors. Our results not only provide valuable information about the expression pattern of transcription factors in different cell types of adult peripheral nerves but also facilitate future studies to understand the function of key transcription factors in the peripheral nerve homeostasis and disease.

## Introduction

The human nervous system is comprised of two parts: the central nervous system (CNS) and the peripheral nervous system (PNS). The PNS consists of the nerves and the ganglia outside the brain and the spinal cord. The main function of the PNS is to connect the CNS to the limbs and organs and to serve as a relay system between the CNS and the rest of the body. The PNS is divided into the somatic nervous system and the autonomic nervous system. The somatic nervous system transmits signals from the brain to organs and striated muscles and is associated with the voluntary control of body movements *via* skeletal muscles. The somatic nervous system includes the sensory nervous system that transmits signals from senses, such as taste, touch, and pain to the spinal cord and the brain. The autonomic nervous system acts largely unconsciously to regulate the physiological functions of the internal organs, such as the heart rate, digestion, respiratory rate, pupillary response, urination, and sexual arousal.

The integrity and function of the PNS is maintained through tight regulation of gene expression in both neurons and supporting non-neuronal cells. Transcription factors (TFs) play a key role in regulating gene expression in the PNS, for example, the myelin gene expression in Schwann cells of adult peripheral nerves is controlled by the TF Egr2. Myelin expression is required for Schwann cells to insulate axons, enabling rapid action potential conduction along the nerves. Mutations that disrupt the Egr2 gene are associated with hereditary myelinopathies (Warner et al., 1998, 1999; Vandenberghe et al., 2002). TFs recognize and bind to specific DNA sequences in the promoter and enhancer regions of target genes and regulate gene transcription through their interaction with other cofactors and RNA polymerase. TFs constitute the second largest gene family in the mammalian genome and not only play essential roles in embryonic development but also drive various biological processes in tissue identity maintenance (Zhou et al., 2017; Lambert et al., 2018).

Currently, the expression pattern of TFs in the cells of adult peripheral nerves has not been systematically analyzed. Creating a cell-type specific TF expression map for the mouse sciatic nerve could facilitate our understanding of the key functions of transcription factors in peripheral nerve homeostasis and disease. Previous gene expression profiling studies using complementary DNA (cDNA) microarrays and bulk mRNA sequencing technologies have identified genes including TFs that are expressed in the intact mouse sciatic nerve (Barrette et al., 2010; Arthur-Farraj et al., 2012; Clements et al., 2017; Pan et al., 2017; Norrmen et al., 2018). However, cDNA microarrays and bulk messenger RNA (mRNA) sequencing technologies are unable to map cell-type specific gene expression profiles. Therefore, immunohistochemistry or *in situ* hybridization techniques are often used to identify gene expression in specific cell types. Recent advances in single-cell RNA sequencing technologies and the development of bioinformatics pipelines have enabled us to rapidly identify the gene expression profile of large numbers of cells in complex tissues comprising of multiple heterogeneous cell populations (Chen et al., 2019). Single-cell RNA sequencing (scRNA-seq) technology has been widely used in different research fields to reveal complex and rare cell populations, map cell-type specific gene expression profiles, and uncover regulatory relationships between genes (Hwang et al., 2018). This technology has only recently been applied to study the cell types and gene expression profiles of cells in the mouse sciatic nerve (Carr et al., 2019; Kalinski et al., 2020; Toma et al., 2020; Wolbert et al., 2020; Ydens et al., 2020). So far, only the expression profile of extracellular signaling molecules has been mapped to specific cell types of intact and injured mouse sciatic nerve (Toma et al., 2020).

One of our research interests is to study the function of TFs in peripheral nerve development and regeneration (Parkinson et al., 2004, 2008; Doddrell et al., 2012, 2013; Mindos et al., 2017; Roberts et al., 2017; Dun et al., 2019). In this report, we reanalyzed the recently published single-cell RNA sequencing dataset for the intact mouse sciatic nerve (Toma et al., 2020) to investigate the cell-type specific expression of TFs in the intact mouse sciatic nerve. We identified 238 TFs expressed in intact mouse sciatic nerves and mapped their cell-type specific expression. We also performed a TF coexpression regulatory network to determine the cell-type specific activity of TFs and their potential downstream targets. Our TF expression and activity map could not only provide valuable information about which TFs are active in a particular cell type but could also provide information about what function the TF has in the adult peripheral nerves. These findings could facilitate future studies to understand the function of key transcription factors in peripheral nerve homeostasis and disease.

## Materials and Methods

### Computational Analysis of Single-Cell and Bulk RNA Sequencing Data Sets

Single-cell RNA-sequencing data set, GSE147285 for intact mouse sciatic nerve was downloaded from the NCBI GEO database (Toma et al., 2020). The GSE147285 dataset was analyzed using the Seurat v.3.2.1^1^ and SC Transform v.0.3 R packages using R v.4.0.2. Quality control plots of number of features, counts, and percentage of mitochondrial content per cell were plotted for each data set and were used to determine the filtering conditions. For the quality control, the cells were filtered using the following conditions: number of features per 200–6,000 cell and percent of mitochondrial DNA content per cell < 8%. Filtered cell data were normalized, variable genes were identified, and the data were scaled using the SCTransform, a recently published highly effective method for removing the technical artifacts from scRNAseq data while retaining the biological heterogeneity (Hafemeister and Satija, 2019). The dimensionality of the dataset was determined using elbow plots to identify the appropriate number of principal components used for clustering. Cell clustering was performed using the FindNeighbors and FindCluster functions in Seurat. Differentially expressed genes (DEGs) were identified using the FindAllMarkers Seurat function using the Wilcoxon rank-sum test for genes with a minimum of 0.25 log-fold change between clusters and expressed in at least 10% of cells between clusters. To annotate the clusters, genes differentially expressed in a one vs. all cluster comparisons were queried for known expression in a literature search and gene expression was plotted. Cell clustering was visualized using t-distributed stochastic neighbor embedding (tSNE) using the FeaturePlot function in Seurat. The t-SNE gene expression overlays, violin plots, dot plots, and heatmaps for cell-type specific marker genes were also plotted using Seurat specific functions. Transcription factor network analysis was performed on the GSE147285 dataset using the R package SCENIC (single-cell regulatory network inference and clustering) v.1.2.0^2^ (Aibar et al., 2017). Transcription factor network plots were visualized using Cytoscape v. 3.8.2 (Shannon et al., 2003) and arranged using the Files Layout Algorithms app v. 1.1.1. Relative micro-RNA (mRNA) expression levels were analyzed from the bulk mRNA sequencing data set GSE108231 (Norrmen et al., 2018).

### Marker Genes for the Identification of Cell Clusters

Cell clusters were identified based on the use of the following established marker genes for cell types of mouse sciatic nerves ([Bibr B21][Bibr B60]; Carr et al., 2019; Renthal et al., 2020; Toma et al., 2020; Wolbert et al., 2020; Xie et al., 2020; Ydens et al., 2020; Chen et al., 2021): Egfl7, Ecscr, Pecam1/Cd31, Tie1, Emcn, Cdh5, and Esam for endothelial cells; Des, Tpm2, Myh11, Acta2, Mylk, Myom1, and Myocd for vascular smooth muscle (VSM) cells; Rgs5, Kcnj8, and Pdgfrb for pericytes; Sox10, Plp1, and S100b for Schwann cells; Cdh2 and L1cam for non-myelinating Schwann cells (nmSCs); Mbp, Mpz, Mag, and Egr2 for myelinating Schwann cells (mSCs): Dcn, Mfap5, Serpinf1, and Gsn for fibroblasts; Sfrp2, Dpt, Pcolce2, Adamts5, Pi16, Sfrp4, Prrx1, Comp, and Ly6c1 for epineurial fibroblasts; Cldn1, Lypd2, Ntn4, Msln, Ntng1, Slc2a1, and Mpzl2 for perineurial cells; Sox9, Osr2, Abca9, Cdkn2a, Cdkn2b, and Plxdc1 for endoneurial fibroblasts; Ptprc and Cd52 as general marker for immune cells.

## Results

### Identification of Transcription Factors Expressed in Intact Mouse Sciatic Nerve

**FIGURE 1:**
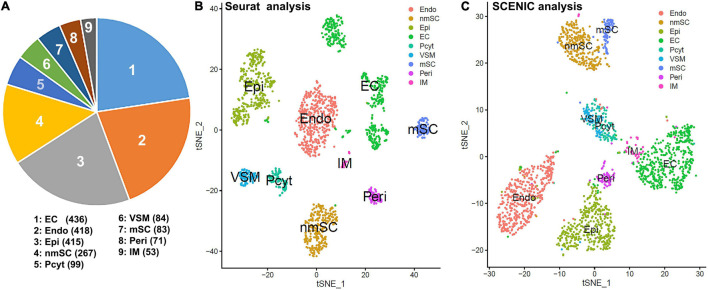
Single-cell transcriptomics defines cellular phenotypes in intact mouse sciatic nerve by both Seurat and single-cell regulatory network inference and clustering (SCENIC) analysis using scRNA-seq data set GSE147285. **(A)** Cell number for endothelial cells (ECs), endoneurial (Endo), perineurial (Peri), and epineurial (Epi) fibroblasts, myelinating Schwann cells (mSCs), and non-myelinating Schwann cells (nmSCs), vascular smooth muscle (VSM) cells, pericytes (Pcyt), and immune (IM) cells. **(B)** T-distributed stochastic neighbor embedding (tSNE) visualization of cell clusters in intact mouse sciatic nerve following Seurat analysis; nine cell clusters have been identified in this analysis based on gene expression. **(C)** The tSNE visualization of transcription factor (TF) regulon activity in intact mouse sciatic nerve following SCENIC re-analysis. Cell types are annotated using the Seurat clustering from gene expression.

To generate an unbiased cellular map for the intact mouse peripheral nerve at single cell resolution, we first analyzed the scRNA-seq data set, GSE147285 for the intact mouse sciatic nerve with the R-package Seurat v3.2.1 in R v.4.0.2 (Toma et al., 2020; Chen et al., 2021). Following quality control and filtration of low-quality cells, a total of 1,926 cells were used in the subsequent analysis. Clustering of cells was based on gene expression using Seurat-identified clusters of mSCs and nmSCs, epineurial, perineurial, and endoneurial fibroblasts, endothelial cells (ECs), VSM cells, pericytes, and immune cells (Figures 1A,B). To study the transcription factor activity in the peripheral nerves during homeostasis, we performed single-cell transcription factor regulatory network inference and clustering using the R-package SCENIC v.1.2.0. Clustering of cells based on SCENIC regulon activity shows that most cell clusters identified by Seurat form distinct groups on the t-SNE plot (Figure 1C). Interestingly, the three sub-groups of ECs that separate on the t-SNE plot based on gene expression actually group together, when clustered, based on the regulon activity (Figure 1C). Figure 1C shows the position of the mSCs and nmSCs that are in closer proximity when considering the regulon activity, and also the epineurial and perineurial fibroblasts and VSM and pericyte cells clusters (Figure 1C). The closer arrangement of subclusters of Schwann cells, fibroblasts, and ECs by SCENIC analysis indicates that common transcriptional activities could exist in the same cell type although they form distinct subclusters with Seurat analysis. In total, we identified 238 TFs that show expression in different cell types by SCENIC analysis (Supplementary Figures 1, 2). We created a cell-type specific expression map for the identified 238 transcription factors (Supplementary Figures 3, 4 and Supplementary Table 1), and investigated their prevalence (percentage of cells expressing TF) and average expression levels in each cell cluster (Supplementary Table 1).

**FIGURE 2:**
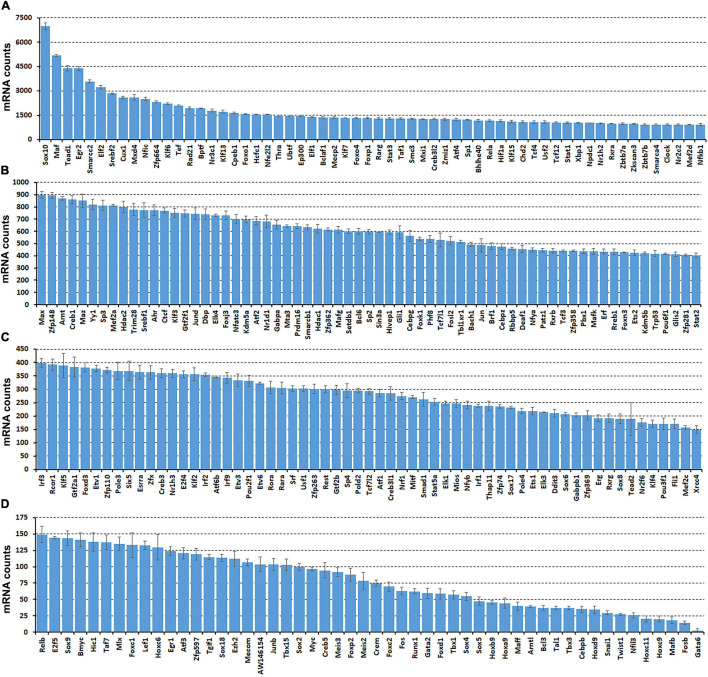
Comparison of normalized messenger RNA (mRNA) counts for the identified 238 TFs in intact mouse sciatic nerve to show their relative expression in mRNA levels. Bulk mRNA sequencing data was analyzed with the data set, GSE108231. **(A)** Sixty TFs have mRNA count bigger than 900. **(B)** Sixty-five TFs have mRNA counts ranging from 400 to 900. **(C)** Sixty-one TFs have mRNA count ranging from 150 to 400. **(D)** Fifty-two TFs have mRNA count smaller bigger than 150. Normalized mRNA counts are shown as mean ± SEM, *n* = 3.

While scRNA-seq data sets are available for the intact mouse sciatic nerve, it takes several hours to obtain single cell suspensions from peripheral nerves for subsequent analysis (Carr et al., 2019; Toma et al., 2020; Wolbert et al., 2020). The *in vitro* tissue enzyme digestion and single cell separation procedures could therefore alter the levels of *in vivo* gene expression (Vanlandewijck et al., 2018; Xu et al., 2019). To confirm that the transcription factors identified by scRNA-seq are expressed in the sciatic nerve and not induced by the preparation of the single cell suspension, we also analyzed the bulk RNA-seq dataset, GSE108231 to compare the relative mRNA levels for the identified 238 TFs in intact mouse sciatic nerve (Norrmen et al., 2018). Analyzing the bulk mRNA-seq data set revealed that all 238 TFs are expressed in the sciatic nerve (Figures 2A–D and Supplementary Table 3). TFs, such as Sox10, Maf, Tead1, Egr2, and Cux1 are highly expressed in the intact mouse sciatic nerve (Figure 2A). Next, we examined their cell-type specific expression pattern using the cell clusters identified by Seurat.

### Transcription Factors Expressed in Schwann Cells

Schwann cells are the most abundant cells in the peripheral nerves, and adult nerve contains both mSCs and nmSCs (Carr and Johnston, 2017; Stierli et al., 2018, 2019). Clustering of cells based on gene expression grouped mSCs and nmSCs into two distinct clusters (Figure 1B). Although a previously study showed that more than 50% of cells are mSCs in the intact mouse sciatic nerve (Stierli et al., 2018), our analysis showed that mSCs form the seventh largest cell cluster (Figures 1A–C) (4.3% of all cells in the analysis). This could be because of the fact that myelin removal beads were used during the preparation of the single-cell suspension for subsequent mRNA extraction, and therefore a large number of mSCs could have been removed (Carr et al., 2019; Wolbert et al., 2020). The nmSCs form the fourth largest cell cluster in the intact mouse sciatic nerve (Figures 1A–C).

**FIGURE 3:**
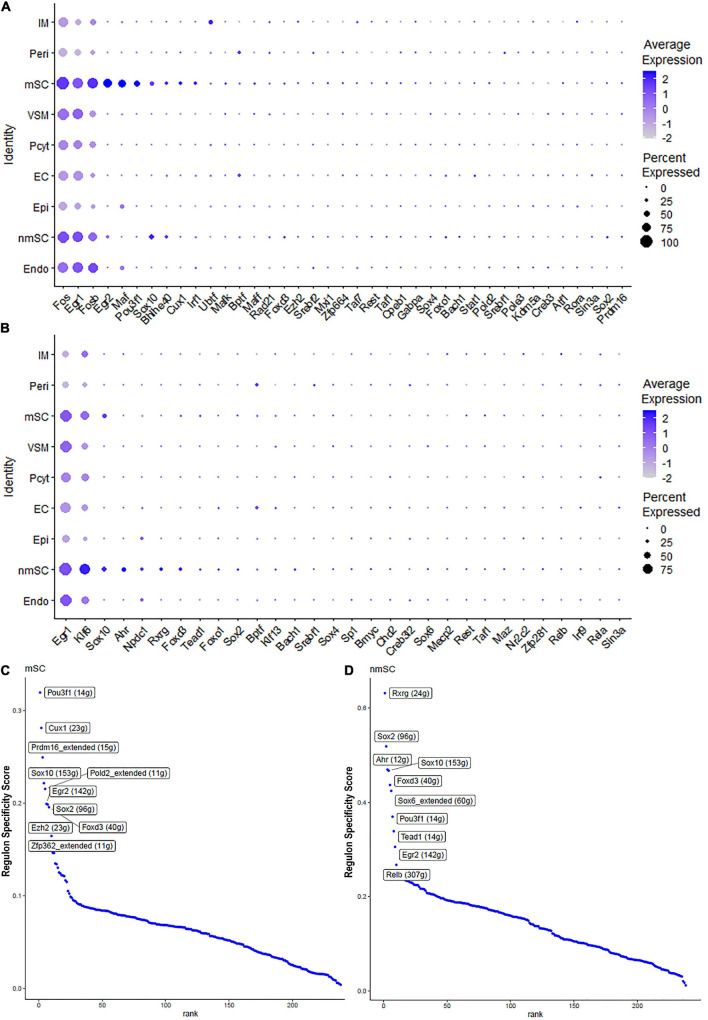
TF expression in mSCs and nmSCs. **(A)** Thirty-nine identified TFs in mSCs showing more than 5% of mSC expression. **(B)** Thirty identified TFs in nmSC showing more than 5% of nmSC expression. **(C)** Top 10 transcription activities in mSCs. **(D)** Regulon-specificity score in nmSCs with top 10 specific TFs labeled.

**FIGURE 4:**
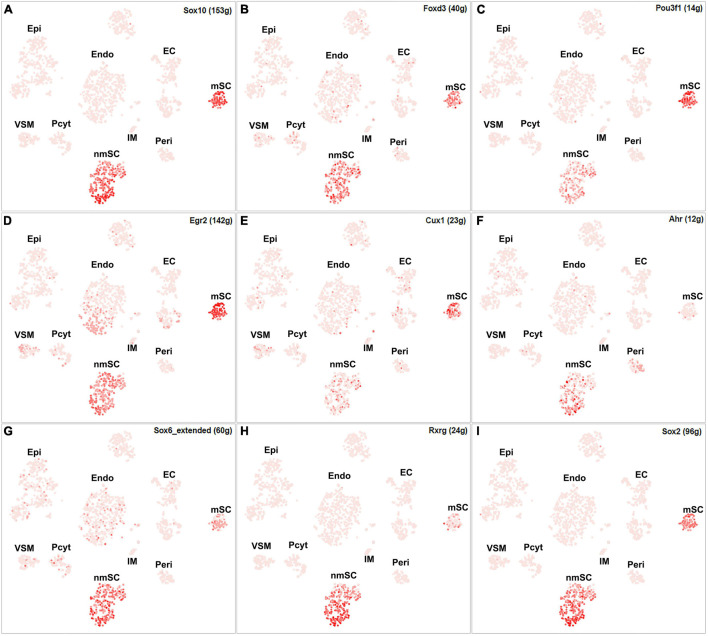
tSNE showing regulon activity for TFs in mSCs and nmSCs. **(A)** Sox10, **(B)** Foxd3, **(C)** Pou3f1, **(D)** Egr2, **(E)** Cux1, **(F)** Ahr, **(G)** Sox6, **(H)** Rxrg and **(I)** Sox2.

Single-cell regulatory network inference and clustering analysis based on mRNA expression in the mSC cluster revealed that 61 TFs show extended regulon activities in mSCs (Supplementary Figure 1 and Supplementary Table 2) and of which 23 TFs are expressed in more than10% of mSC (Figure 3A and Supplementary Table 2). The top 10 TFs specific to mSC include Pou3f1, Cux1, Prdm16, Sox10, Pold2, Egr2, Sox2, Foxd3, Ezh2, and Zfp362 (Figure 3C). There are 53 TFs showing extended regulon activities in nmSCs (Supplementary Figure 1 and Supplementary Table 2), and 15 TFs are expressed in more than 10% of nmSC (Figure 3B and Supplementary Table 2). Top 10 TF activities in nmSC include Rxrg, Sox2, Ahr, Sox10, Foxd3, Sox6, Pou3f1, Tead1, Egr2, and Relb (Figure 3D). Sox10 and Foxd3 are exclusively expressed in both mSCs and nmSCs and both have strong transcription activities in mSCs and nmSCs (Figures 3A–D, 4A,B). Previous studies confirmed that Sox10 and Foxd3 are expressed in neural crest cells, and both are required to specify SC lineage from neural crest cells (Jessen and Mirsky, 2019). Their exclusive expression in both mSCs and nmSCs of the adult nerve indicates that they are also required to maintain the SC phenotype in adult peripheral nerves.

Although SCENIC analysis indicated that Egr2 and Pou3f1 have transcription activities in both mSCs and nmSCs (Figures 3C,D, 4C,D), Egr2 and Pou3f1 are highly expressed in the mSC cluster but their expression in the nmSC cluster is low (Figures 3A,B and Supplementary Tables 1, 2). Previous studies demonstrated that Egr2/Krox20 and Pou3f1/Oct6 are required to initiate myelination during the peripheral nerve development and maintain myelin gene expression in the mSCs of adult peripheral nerves (Parkinson et al., 2004; Jessen and Mirsky, 2005). In contrast, Cux1 shows exclusive expression in mSCs (Figure 4E), while Ahr, Sox6, and Rxrg have exclusive expression in nmSCs (Figures 4F–H). Bulk RNA-seq analysis revealed that Cux1 has a higher level of expression in the intact mouse sciatic nerve (Figure 2A). Therefore, it is interesting to study Cux1 function in the mSCs of adult peripheral nerves. SCENIC analysis indicated that Sox2 is also active in both mSCs and nmSCs (Figure 4I). However, our previous studies confirmed that Sox2 is not expressed in mSCs of the intact nerve but it is upregulated in SCs following peripheral nerve injury (Roberts et al., 2017; Dun et al., 2019); thus Sox2 expression in mSC indicates that Sox2 could be upregulated in mSCs during the single-cell suspension preparation. Overexpression of Sox2 in mSCs inhibits myelination (Doddrell et al., 2013; Roberts et al., 2017), indicating that Sox2 expression in nmSC is required to maintain the non-myelinating phenotype of nmSC.

### Transcription Factors Expressed in Fibroblasts

**FIGURE 5:**
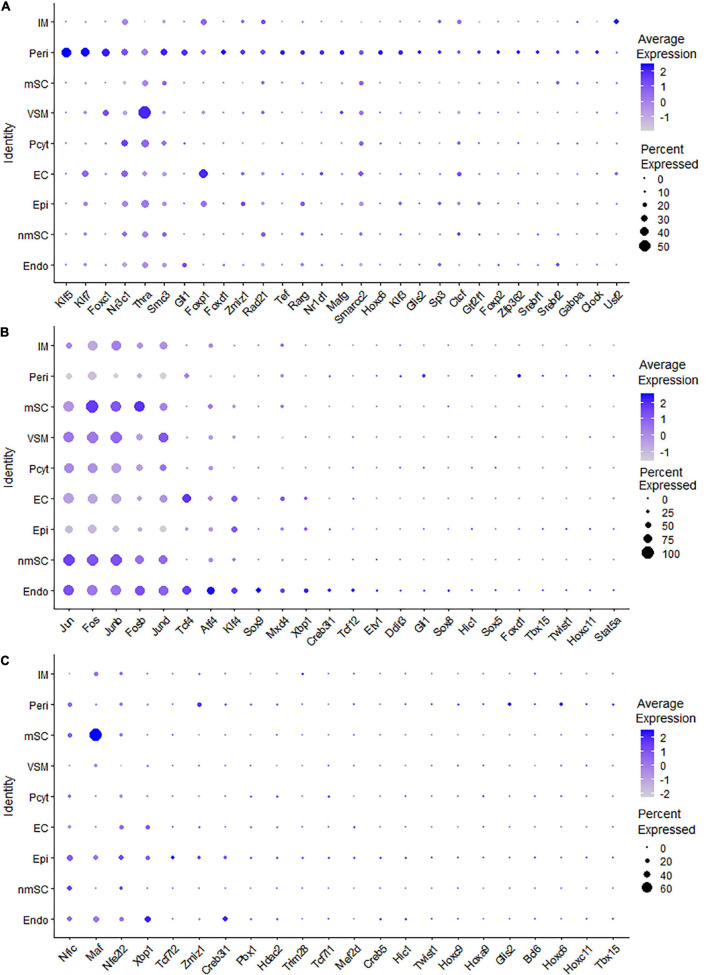
TF expression in fibroblasts. **(A)** Twenty-nine identified TFs in perineurial fibroblasts expressed in more than 10% of perineurial fibroblast cells. **(B)** Twenty-four identified TFs in endoneurial fibroblasts expressed in more than 5% of endoneurial fibroblast cells. **(C)** Twenty-two identified TFs in epineurial fibroblasts expressed in more than 5% of epineurial fibroblast cells.

**FIGURE 6:**
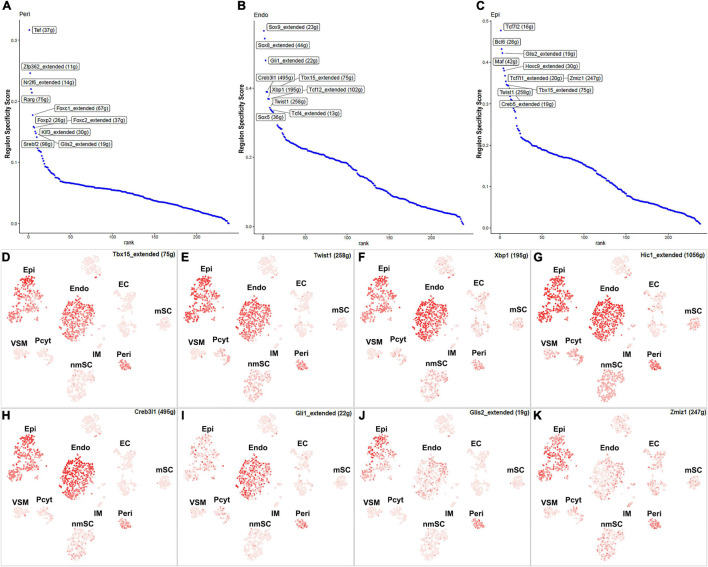
TFs showing regulon activities in fibroblasts. **(A)** Regulon-specificity score in perineurial fibroblasts with top 10 specific TFs labeled. **(B)** Regulon-specificity score in endoneurial fibroblasts with top 10 specific TFs labeled. **(C)** Regulon specificity score in epineurial fibroblasts with top 10 specific TFs labeled. **(D–K)** tSNE showing regulon activity for Tbx15, Twist1, Xbp1, Hic1, Creb3l1, Gli1, Glis2, and Zmiz1 in fibroblasts.

Fibroblasts are the second most abundant cells in adult peripheral nerves; they localize in the endoneurium, perineurium, and epineurium to provide structural support, create regional separation, and protect nerve fibers from damage (Parrinello et al., 2010; Stierli et al., 2018; Carr et al., 2019; Diaz-Flores et al., 2020). Cell clustering based on gene expression identified the following three fibroblast sub-types: endoneurial, perineurial, and epineurial fibroblasts (Figures 1B,C). Perineurial fibroblasts have the highest number of extended regulon activities compared to endoneurial and epineurial fibroblasts (Supplementary Table 2). Forty-eight TFs show extended regulon activities in perineurial fibroblasts (Supplementary Figure 1 and Supplementary Table 2), with 29 of them expressed in at least 10% of perineurial fibroblasts (Figure 5A). The top 10 active transcription factors specific to perineurial fibroblasts include Tef, Zfp362, Nr2f6, Rarg, Foxc1, Foxp2, Foxc2, Klf3, Glis2, and Srebf2 (Figure 6A). Twenty-five TFs have extended regulon activities in endoneurial fibroblasts (Supplementary Figure 1 and Supplementary Table 2), 19 of them are expressed in more than 10% of endoneurial fibroblast cells (Figure 5B). The top 10 specific TFs active in endoneurial fibroblasts include Sox9, Sox8, Gli1, Creb3l1, Tbx15, Xbp1, Tcf12, Twist1, Tcf4, and Sox5 (Figure 6B). Twenty-three TFs have extended regulon activities in epineurial fibroblasts (Supplementary Figure 1 and Supplementary Table 2) and 11 of them show expression in 10% of endoneurial fibroblast cells (Figure 5C). The top 10 active TFs specific to epineurial fibroblasts include Tcf7l2, Bcl6, Glis2, Maf, Hoxc9, Tcf7l1, Zmiz1, Twist1, Tbx15, and Creb5 (Figure 6C).

**FIGURE 7:**
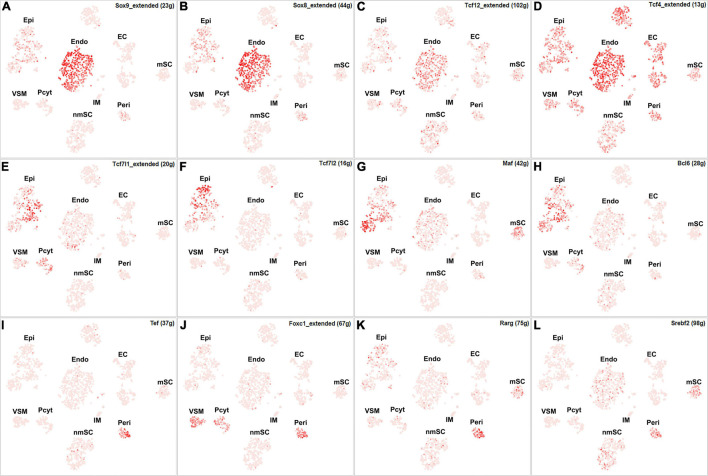
tSNE showing regulon activity for TFs in fibroblasts. **(A)** Sox9, **(B)** Sox8, **(C)** Tcf12, **(D)** Tcf4, **(E)** Tcf7l1, **(F)** Tcf7l2, **(G)** Maf, **(H)** Bcl6, **(I)** Tef, **(J)** Foxc1, **(K)** Rarg and **(L)** Srebf2.

Among all the TFs identified in endoneurial, perineurial, and epineurial fibroblasts, Tbx15 shows high transcriptional activity in all the three clusters of fibroblasts (Figure 6D). Twist1, Xbp1, Hic1, and Creb3l1 have transcriptional activity in both perineurial and epineurial fibroblasts (Figures 6E–H). Gli1 is active in both endoneurial and perineurial fibroblasts (Figure 6I). Glis2 and Zmiz1 have transcriptional activity in both perineurial and epineurial fibroblasts (Figures 6J,K). Sox9, Sox8, Tcf12, and Tcf4 have transcriptional activity in endoneurial fibroblasts (Figures 7A–D). Previously, Sox9 has been used as a marker gene in scRNA-seq data studies to identify the endoneurial fibroblast clusters (Carr et al., 2019; Toma et al., 2020). Tcf7l1, Tcf7l2, Maf, and Bcl6 are active in epineurial fibroblasts (Figures 7E–H). Tef, Rarg, Foxc1, and Srebf2 have transcriptional activity in perineurial fibroblasts (Figures 7I–L). This analysis revealed that endoneurial, perineurial, and epineurial fibroblasts not only share some common fibroblast lineage transcription factor activities but also have distinct cell-type specific transcriptional activities, which might drive the expression of key genes not only to preserve their cell identity but also to maintain distinct functions in adult peripheral nerves.

### Transcription Factors Expressed in Cells Associated With Peripheral Nerve Blood Vessels

**FIGURE 8:**
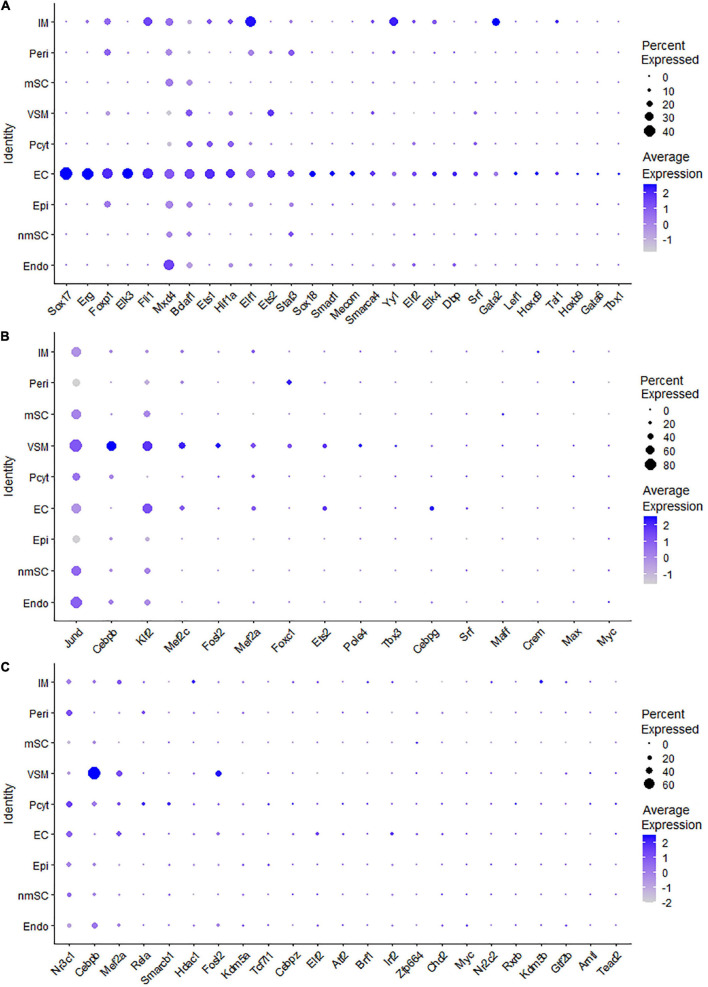
TF expression in ECs, VSM cells, and pericytes. **(A)** Twenty-eight identified TFs in ECs expressed in more than 5% of ECs. **(B)** Sixteen identified TFs in VSM cells expressed in more than 5% of VSM cells. **(C)** Twenty-three identified TFs in pericytes expressed in more than 5% of pericyte cells.

**FIGURE 9:**
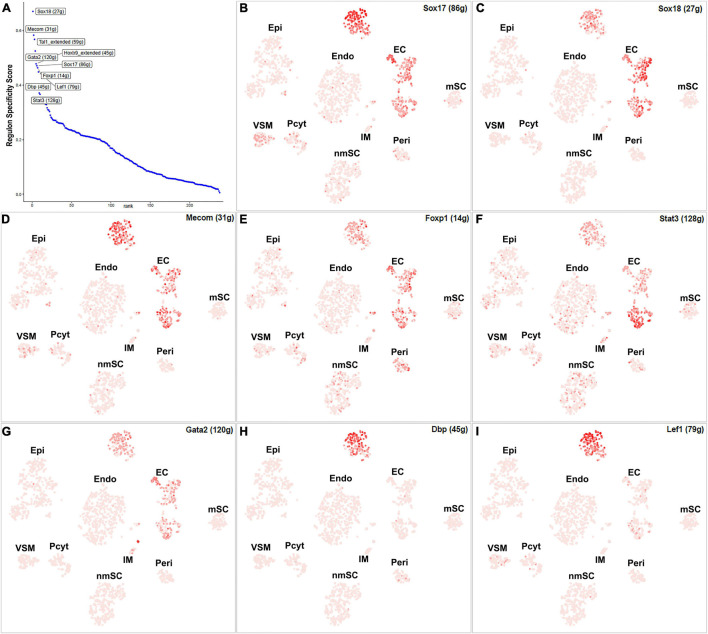
TF activity in ECS. **(A)** Regulon-specificity score in ECs with top 10 specific TFs labeled. **(B–I)** The tSNE showing regulon activity for Sox17, Sox18, Mecom, Foxp1, Stat3, Gata2, Lef1, and Dbp in endothelial cells.

Endothelial cells of the blood vessels show remarkable heterogeneity and their phenotypes vary in time and space, differ in structure and function, and change in health and disease (Aird, 2012). Twenty-eight TFs show extended regulon activity in the EC cluster (Supplementary Figure 1 and Supplementary Table 2), and 23 of them are expressed in more than 10% of ECs (Figure 8A). Sox18, Mecom, Tal1, Gata2, Hoxb9, Sox17, Foxp1, Lef1, Dbp, and Stat3 are the top 10 specific TFs active in ECs of the intact mouse sciatic nerve (Figure 9A). Sox17, Sox18, Mecom, Foxp1, Stat3, and Gata2 have high expression in all three EC sub-clusters (Figures 9B–G). In contrast, Dbp and Lef1 show expression only in one of the three EC sub-clusters (Figures 9H,I).

**FIGURE 10:**
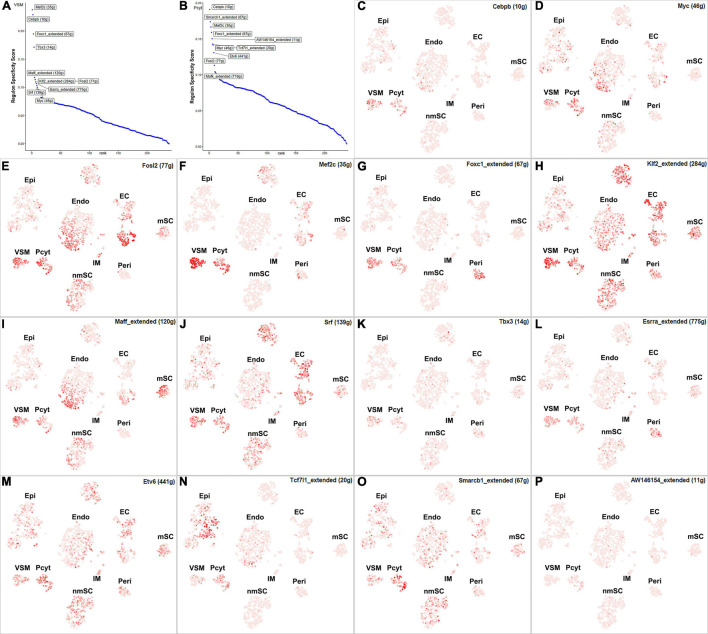
TF activity in VSM cells and pericytes. **(A)** Regulon-specificity score in VSM cells with top 10 specific TFs labeled. **(B)** Regulon-specificity score in pericytes with top 10 specific TFs labeled. **(C–P)** The tSNE showing regulon activity for Cebpb, Myc, Fosl2, Mef2c, Foxc1, Klf2, Maff, Srf, Tbx3, Esrra, Etv6, Tcf7l1, Smarcb1, and AW146154 in VSM cells and pericytes.

The blood vessels also include VSM cells and pericytes (Carr et al., 2019; Toma et al., 2020; Wolbert et al., 2020). While 17 TFs have been identified with extended regulon activity in VSM (Supplementary Figure 1 and Supplementary Table 2), 12 of them show expression in more than 10% of VSM cells (Figure 8B). The top 10 specific TFs active in VSM include Mef2c, Cebpb, Foxc1, Tbx3, Maff, Klf2, Fosl2, Esrra, Srf, and Myc (Figure 10A). Thirty-one TFs have been identified with extended regulon activity in pericytes (Supplementary Figure 1 and Supplementary Table 2), and nine of them show expression in more than 10% of pericyte cells (Figure 8C). The top 10 specific TFs active in pericytes include Cebpb, Smarcb1, Mef2c, Foxc1, AW146154, Tcf7l1, Myc, Etv6, Fosl2, and Mafk (Figure 10B). Mef2c, Cebpb, Foxc1, Fosl2, and Myc have expression in both VSM and pericyte clusters (Figures 10C–G), indicating that VSM cells and pericytes could express similar genes in the intact mouse sciatic nerve. Previous scRNA-seq data analysis also used the same set of marker genes to identify both VSM cell and pericyte clusters (Carr et al., 2019; Toma et al., 2020; Wolbert et al., 2020; Chen et al., 2021). Klf2, Maff, Srf, Tbx3, Esrra, Etv6, Tcf7l1, Smarcb1, and AV146154 also have expression in both VSM and pericyte clusters (Figures 10H–P); however, they also show expression in other cell clusters (Figures 10H–P).

### Transcription Factors Expressed in Immune Cells

**FIGURE 11:**
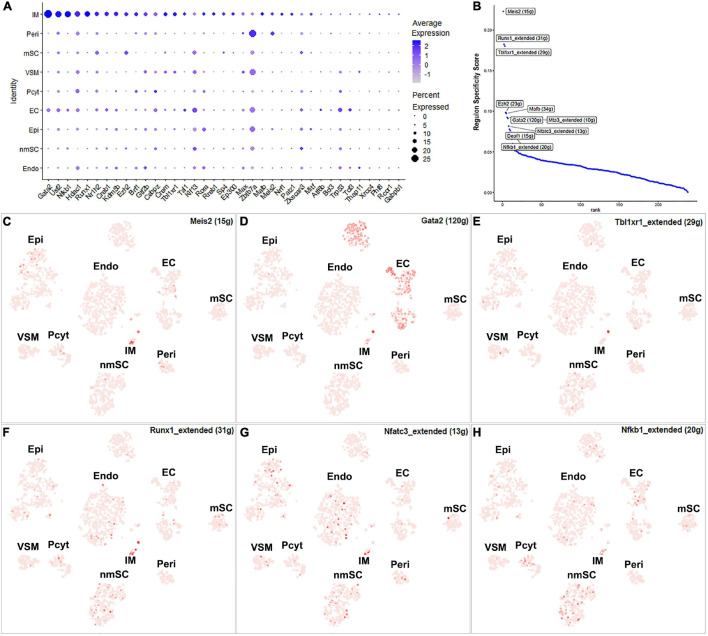
TF activity in immune cells. **(A)** Thirty-seven identified TFs expressed in more than 5% of immune cells. **(B)** Regulon-specificity score in immune cells with top 10 specific TFs labeled. **(C–H)** The tSNE showing regulon activity for Meis2, Gata2, Tbl1xr1, Runx1, Nfatc3, and Nfkb1 in immune cells.

The number of immune cells in the intact nerve is low and all the immune cells have been gathered into one cluster (Figures 1B,C). Previous studies have shown that resident macrophages are the major immune cells in the intact nerve and they comprise 8–9% of cells of the mouse sciatic nerve (Stierli et al., 2018; Amann and Prinz, 2020). Mast cells, B cells, T cells, and NK cells are also present in the intact nerves with low numbers (Wolbert et al., 2020). Immune cells provide protection to the nerves from pathogen infection; they also help to clear cell debris (Rotshenker, 2011; Amann and Prinz, 2020). While 43 TFs have transcription activity in the immune cell cluster, 24 of them are expressed in more than 10% of immune cells (Supplementary Figure 1 and Supplementary Table 2). Meis2, Runx1, Tbl1xr1, Ezh2, Gata2, Mafb, Mta3, Nfatc3, Deaf1, and Nfkb1 are the top 10 specific TFs for immune cells (Figure 11).

## Discussion

Single-cell RNA sequencing technologies not only reveal the cell types present in a particular tissue but they also reveal the gene expression patterns present at the single-cell level (Hwang et al., 2018; Chen et al., 2019). In recent years, scRNA-seq technologies have been widely applied on different tissues of various species to reveal cell types and meaningful gene expression profiles at single-cell resolution (Hwang et al., 2018; Chen et al., 2019). However, this technology has only recently been applied to the mouse sciatic nerve before and after injury (Carr et al., 2019; Toma et al., 2020; Wolbert et al., 2020). A key goal of scRNA-seq data analysis is to reveal gene expression at single-cell resolution (Jaitin et al., 2014; Grun et al., 2015; Chen et al., 2016; Cao et al., 2017; Rosenberg et al., 2018). In this report, we studied cell-type specific expression pattern of TFs in the cells of intact mouse sciatic nerve by re-analysis of the recently published scRNA-seq data set (Toma et al., 2020). We identified 238 TFs that are expressed in different cell types of the mouse sciatic nerve. The VSM cells have the lowest number of transcription factors expressed with 17 transcription factors identified. The mSCs have the highest number of transcription factors expressed with 61 transcription factors identified. The high number of transcription factors expressed in mSCs indicate their central role in the peripheral nerve homeostasis.

A number of scRNA-seq technologies, such as Drop-seq and Smart-seq2 have been developed and these methods possess their unique features with distinct advantages and disadvantages (Hwang et al., 2018; Chen et al., 2019). Due to technical limitations and the complexity of biological factors, the scRNA-seq data are often noisier and more complex than bulk RNA-seq data (Hwang et al., 2018; Chen et al., 2019). Therefore, high-throughput single-nucleus genomics have been developed to profile frozen or hard-to-dissociate tissues (Korrapati et al., 2019; Denisenko et al., 2020; Slyper et al., 2020). This technology can prevent the induction of immediate early gene expression. However, cell nuclei have lower amounts of mRNA compared to cells. It is more challenging to enrich mRNAs for specific cell types of interest (Korrapati et al., 2019; Denisenko et al., 2020; Slyper et al., 2020). Single-nucleus genomics have revealed that, within hours of injury, the upregulation of Atf3 and Jun could be detected in neurons following peripheral nerve injury (Renthal et al., 2020).

Our analysis revealed that the AP-1 TF family members, such as Fos, Fosb, Jun, Junb, and Jund are all highly expressed in the intact mouse sciatic nerve (Figure 5B) and have been shown to be immediate early genes that respond to neuronal activation or tissue injury (Xu et al., 2019). Previous work for single-cell profiling on small vessels in the brain has suggested that immediate early genes, such as Fos, Fosb, Jun, and Junb, are enriched with cells isolated from solid organs or vascular tissue (Vanlandewijck et al., 2018). Jun and Atf3 are important injury-induced TFs involved in activating the neuronal intrinsic regeneration program following axon damage (Tsujino et al., 2000; Parsadanian et al., 2006; Hunt et al., 2012). Early studies have already shown that their expression in the adult peripheral nerve is very low but are rapidly induced in the distal nerve following injury (Arthur-Farraj et al., 2012, 2017; Fontana et al., 2012; Patodia and Raivich, 2012; Blom et al., 2014; Ko et al., 2018). The data set that we used in this study was generated by Drop-seq technology (Toma et al., 2020). Due to the presence of strong connective tissues, such as epineurium and perineurium in intact adult peripheral nerves, and the presence of myelin in mSC, adult peripheral nerves are a challenging tissue to prepare high-quality single cell suspension for subsequent scRNA-seq analysis. The nerve enzyme dissociation procedures and the subsequent single cell separation by drop-seq technology often take several hours in order to extract RNA from prepared single cells (Andersen et al., 2016; Toma et al., 2020). Thus, this immediate early gene signature in intact mouse sciatic nerve likely represents a rapid induction of expression during the process of tissue dissociation and single cell separation.

In summary, we identified 238 TFs that are expressed in cells of intact mouse sciatic nerve. We created a cell-type specific expression map for the identified 238 transcription factors and examined the activity of the TFs in each cell type by building a TF regulatory network from the single-cell RNA-seq data. The cell-type specific expression map not only provides valuable information about TF expression pattern but also indicates the possible function of the TFs in the different cell types of adult peripheral nerves. We believe that our findings could facilitate future studies to understand the function of key transcription factors in peripheral nerve homeostasis and diseases. We also demonstrated that single-cell RNA sequencing technology is an important tool for studying TFs in cells of the peripheral nerves on a large scale.

## Data Availability Statement

The original contributions presented in the study are included in the article/Supplementary Material, further inquiries can be directed to the corresponding author/s.

## Author Contributions

XD designed the research and wrote the manuscript. ML, QM, and MB performed data analysis. All authors contributed to the article and approved the submitted version.

## Conflict of Interest

The reviewer SY declared a shared affiliation, though no other collaboration, with one of the authors XD to the handling Editor. The authors declare that the research was conducted in the absence of any commercial or financial relationships that could be construed as a potential conflict of interest.

## Publisher’s Note

All claims expressed in this article are solely those of the authors and do not necessarily represent those of their affiliated organizations, or those of the publisher, the editors and the reviewers. Any product that may be evaluated in this article, or claim that may be made by its manufacturer, is not guaranteed or endorsed by the publisher.

## Footnotes

1
https://satijalab.org/seurat/

2
https://scenic.aertslab.org

## Supplementary Material

Supplementary Figure 1 Heatmap for the identified 238 TFs to show their cell-type specific activity in different cell types of intact mouse sciatic nerve. This heatmap shows extended regulons that have lower confidence than the non-extended regulons. We include this heatmap to give a broad overview for other scientists if they wish to explore it in the future.

Supplementary Figure 2 Heatmap for non-extended regulons of high confidence TFs to show their cell- type specific activity in different cell types of intact mouse sciatic nerve.

Supplementary Figures 3, 4 Dotplots for the identified 238 TFs to show their expression profile in different cell types of intact mouse sciatic nerve.

Supplementary Table 1 Percentage of expressed cells and average expression level for the identified 238 TFs in each cell type of intact mouse sciatic nerve.

Supplementary Table 2 Average of normalized mRNA counts for the identified 238 TFs in intact mouse sciatic nerve. The mRNA counts were analyzed with bulk mRNA sequencing data set GSE108231, *n* = 3.

Supplementary Table 3 List of TFs showing expression in each cell type of intact mouse sciatic nerve. Percentage of expressed cells and average expression level were analyzed at the same time.
