# Supplementary figures and images for: Single-Cell Regulatory Network Inference and Clustering Identifies Cell-Type Specific Expression Pattern of Transcription Factors in Mouse Sciatic Nerve
Source: Front Cell Neurosci. 2021 Dec 8;15:676515. doi: 10.3389/fncel.2021.676515 (PMC8693779; doi:10.3389/fncel.2021.676515)

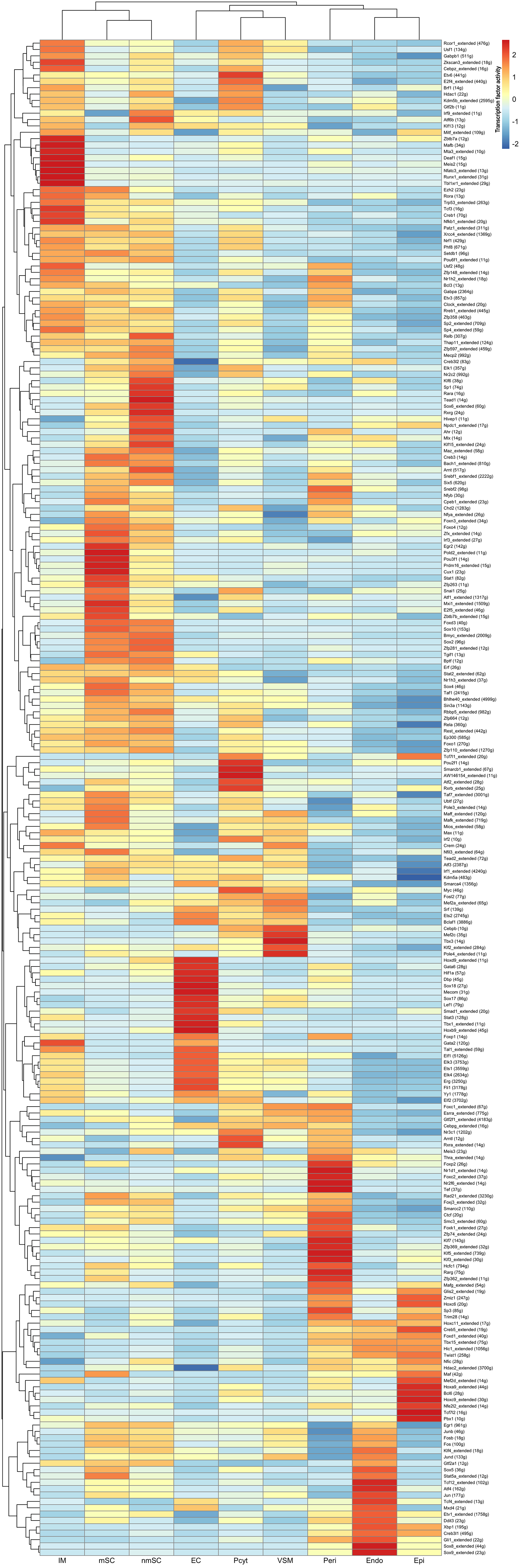

Supplement: Supplementary Figure 1 — Heatmap for the identified 238 TFs to show their cell-type specific activity in different cell types of intact mouse sciatic nerve. This heatmap shows extended regulons that have lower confidence than the non-extended regulons. We include this heatmap to give a broad overview for other scientists if they wish to explore it in the future. [file Image_1.TIF]

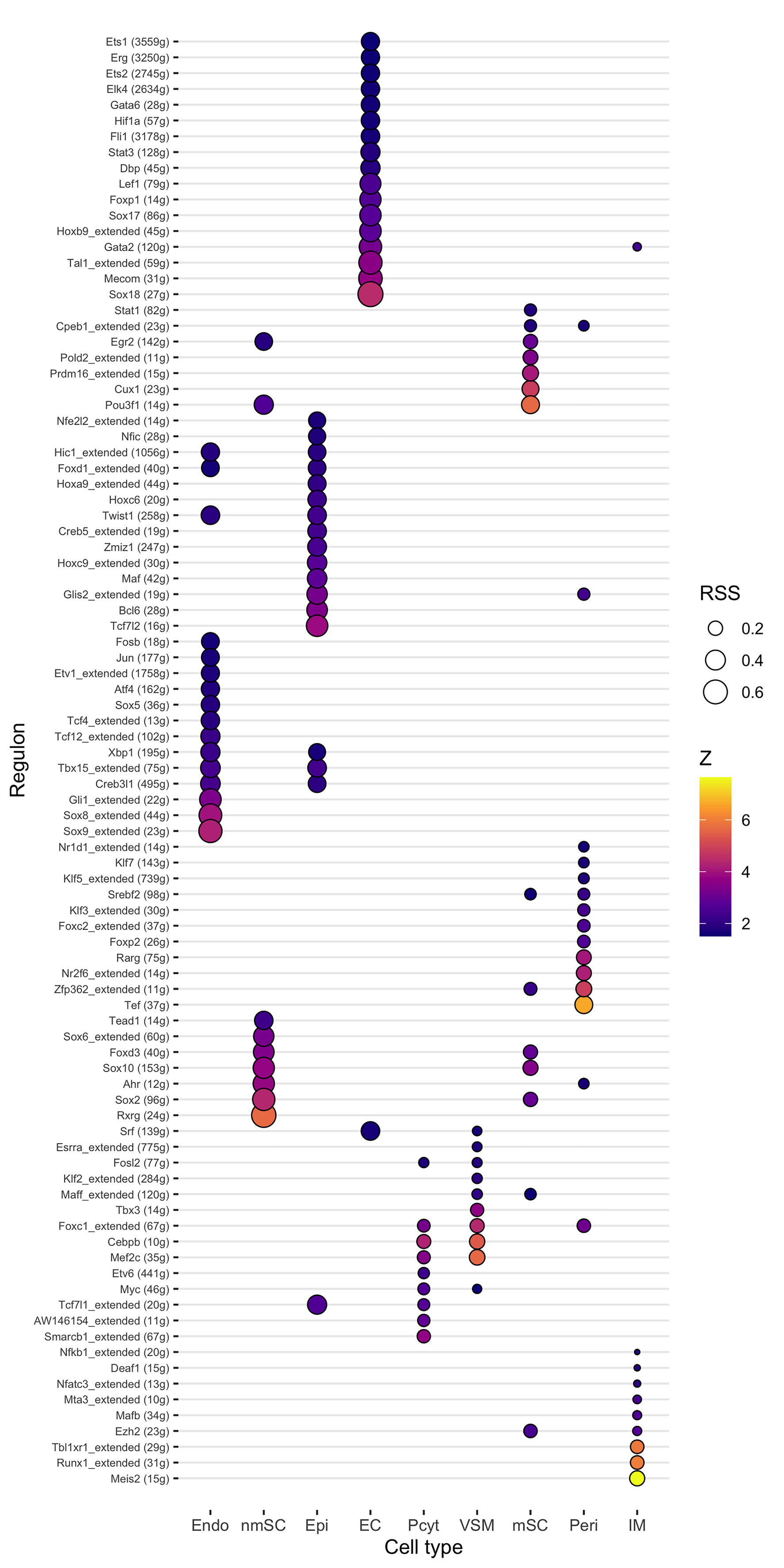

Supplement: Supplementary Figure 2 — Heatmap for non-extended regulons of high confidence TFs to show their cell- type specific activity in different cell types of intact mouse sciatic nerve. [file Image_2.TIF]

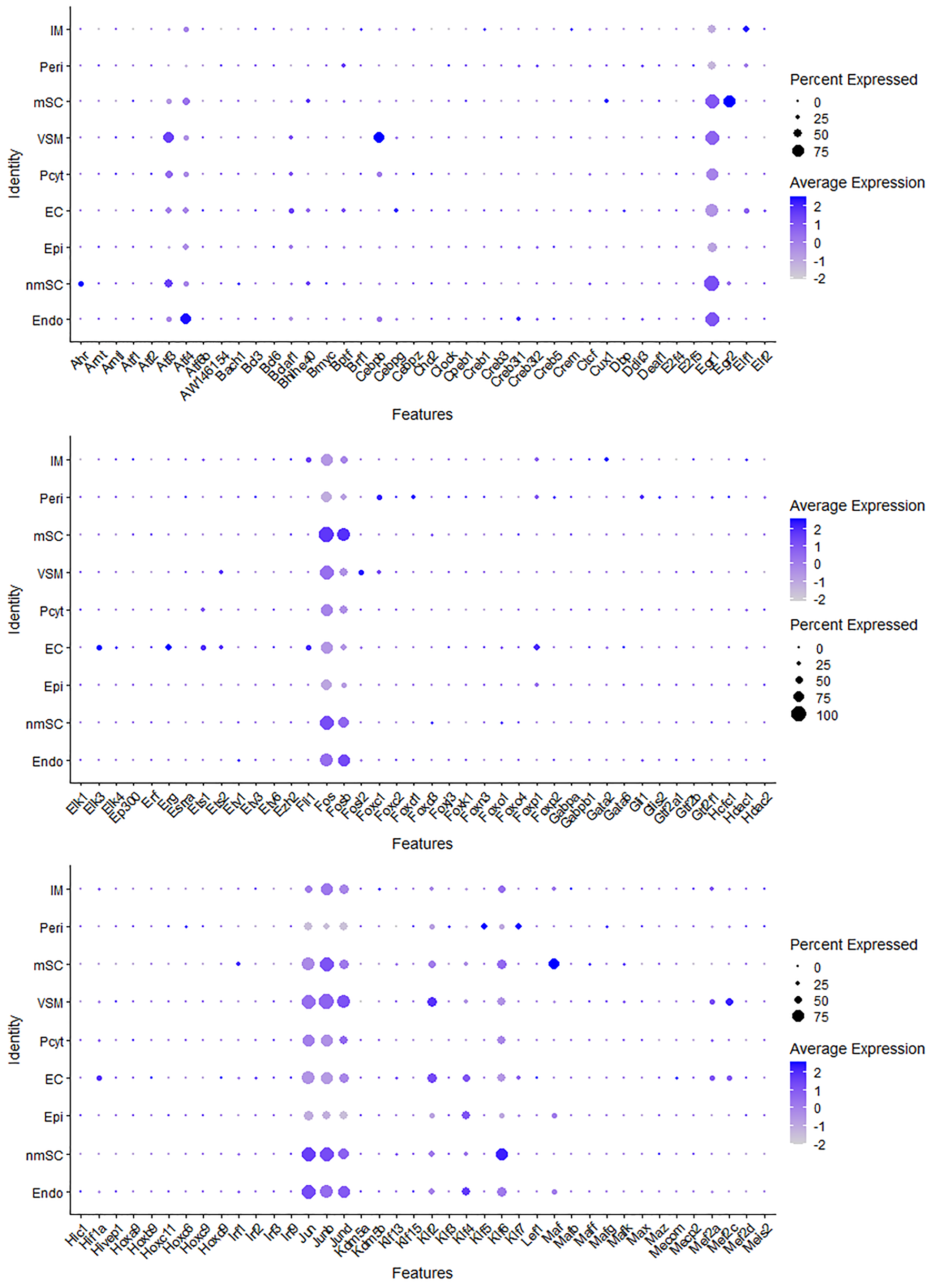

Supplement: Supplementary file 3 [file Image_3.TIF]

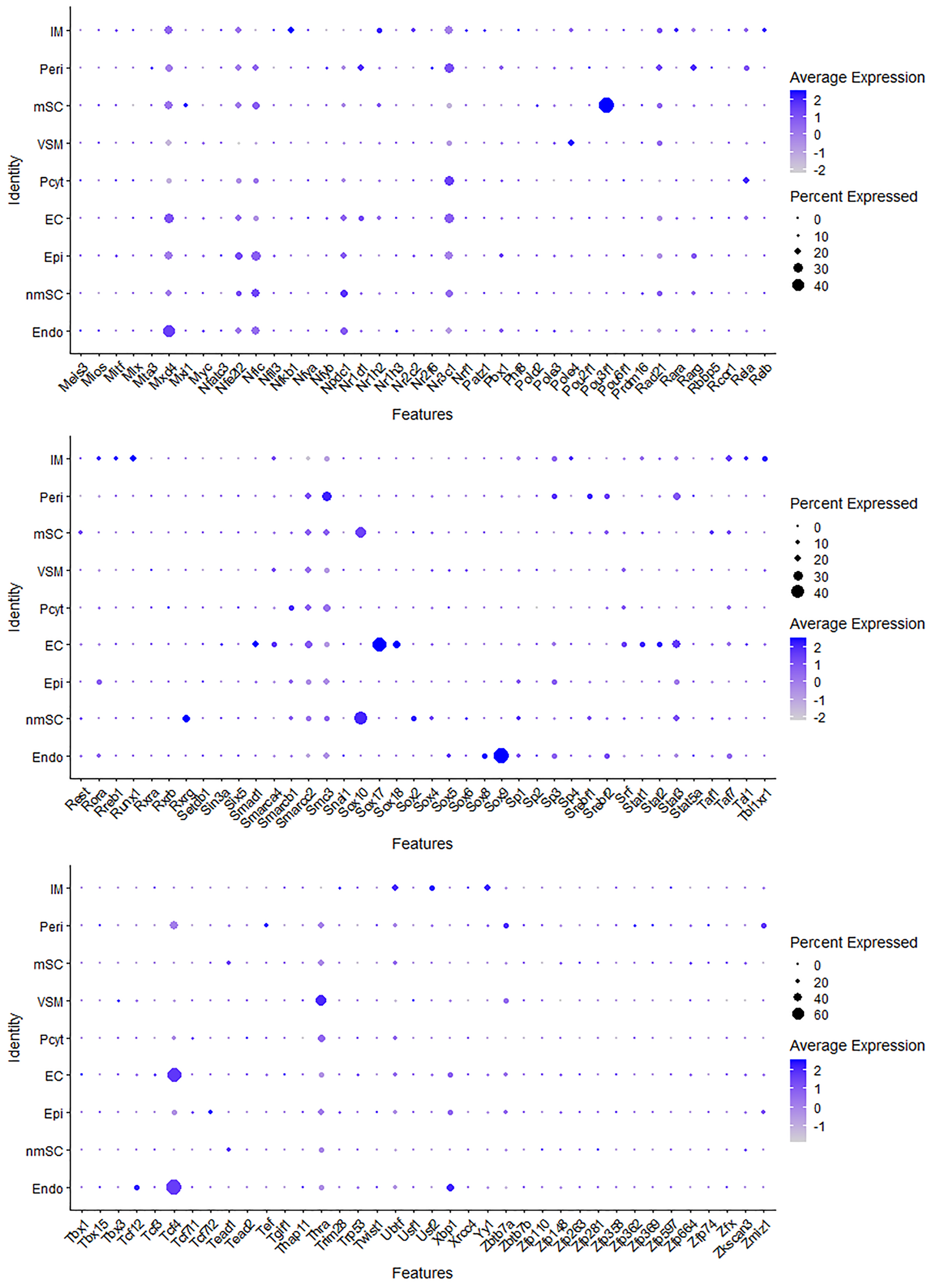

Supplement: Supplementary Figures 3, 4 — Dotplots for the identified 238 TFs to show their expression profile in different cell types of intact mouse sciatic nerve. [file Image_4.TIF]
